# Effect of long-chain omega-3 polyunsaturated fatty acids on cardiometabolic factors in children with acute lymphoblastic leukemia undergoing treatment: a secondary analysis of a randomized controlled trial

**DOI:** 10.3389/fendo.2023.1120364

**Published:** 2023-04-14

**Authors:** Lourdes Barbosa-Cortes, Salvador Atilano-Miguel, Jorge Alfonso Martin-Trejo, Emmanuel Jiménez-Aguayo, Fabian Ismael Martínez-Becerril, Mardia López-Alarcón, Juan Manuel Mejía Aranguré, Jorge Maldonado-Hernández, Suyly Delgadillo-Portillo, Brenda Guzmán-Castro, Jazmín Delgadillo-Portillo, Ana Añoveros-Barrera, Karina Anastacia Solis-Labastida, Benito Alejandro Bautista-Martinez, Azalia Juárez-Moya, Zaira Hernández-Piñón, Laura Eugenia Espinoza Hernández, Nora N. Núñez-Villegas, Elva Jiménez-Hernández, Ruy X. Pérez-Casillas

**Affiliations:** ^1^ Unidad de Investigación Médica en Nutrición, Unidad Médica de Alta Especialidad (UMAE), Hospital de Pediatría, Centro Médico Nacional Siglo XXI, Instituto Mexicano del Seguro Social (IMSS), Ciudad de México, Mexico; ^2^ Servicio de Hematología, Unidad Médica de Alta Especialidad (UMAE), Hospital de Pediatría, Centro Médico Nacional Siglo XXI, Instituto Mexicano del Seguro Social (IMSS), Ciudad de México, Mexico; ^3^ Instituto Nacional de Medicina Genómica, Ciudad de México, Mexico; ^4^ Facultad de Medicina, Universidad Nacional Autónoma de México, Ciudad de México, Mexico; ^5^ Servicio de Hematología Pediátrica, Unidad Médica de Alta Especialidad (UMAE), Hospital General “Dr. Gaudencio González Garza” Centro Médico Nacional la Raza, Instituto Mexicano del Seguro Social (IMSS), Ciudad de México, Mexico

**Keywords:** acute lymphoblastic leukemia, hypertriglyceridemia, atherogenic index of plasma, cytokines, android/gynoid fat, IL-6 (Interleukin 6), ω3-LCPUFAs supplementation

## Abstract

**Introduction:**

Increased triglycerides (TGs) are a major risk factor for cardiovascular disease. Furthermore, hypertriglyceridemia is commonly associated with a reduction of high-density lipoprotein cholesterol (HDL-C) and an increase in atherogenic small-dense low-density lipoprotein (LDL-C) levels. Studies provide support that polyunsaturated omega-3 fatty acids (ω3-LCPUFAs) are cardioprotective and have antithrombotic and anti-inflammatory effects. The potential effects of ω3-LCPUFAs on cardiometabolic factors and anti-inflammatory actions in children with acute lymphoblastic leukemia (ALL) are limited. This is a secondary analysis of a previous clinical trial registered at clinical trials.gov (# NCT01051154) that was conducted to analyze the effect of ω3-LCPUFAs in pediatric patients with ALL who were receiving treatment.

Objective: To examine the effect of supplementation with ω3-LCPUFAs on cardiometabolic factors in children with ALL undergoing treatment.

**Methods:**

Thirty-four children (placebo group: 20 patients; ω3-LCPUFAs group: 14 patients) aged 6.7 ± 2.7 years who were newly diagnosed with ALL were evaluated. Children were randomized to receive either ω3-LCPUFAs or placebo capsules (sunflower oil). ω3-LCPUFAs were administered in the form of 500-mg soft capsules. The ω3-LCPUFA capsules contained 225 mg of DHA, 45 mg of EPA, and 20 mg of another ω3-LCPUFAs. The omega-3 dose was administered at a rate of 0.100 g/kg of body weight/day for three months. Main outcomes: Fasting cholesterol, HDL-C, very-low-density lipoprotein (VLDL-C), TGs, atherogenic index of plasma (AIP), android/gynoid ratio (A/GR), IL-6, TNF-α, and percentage of fat mass (DXA) were measured in all patients. Fatty acid analyses in red blood cells were performed with gas chromatography.

**Results:**

We found significantly lower levels of TGs (p=0.043), VLDL-C (p=0.039), IL-6 (p=0.025), and AIP (p=0.042) in the ω3-LCPUFAs group than in the placebo group at three months. In contrast, the total cholesterol concentration was higher at 3 months in the ω3-LCPUFAs group than in the placebo group (155 mg/dl vs. 129 mg/dl, p=0.009). The number of children with hypertriglyceridemia (85% vs. 50%; p=0.054) tended to be lower between the time of diagnosis and after 3 months of supplementation with ω3-LCPUFAs.

**Conclusion:**

These findings support the use of ω3-LCPUFAs to reduce some adverse cardiometabolic and inflammatory risk factors in children with ALL.

**Clinical trial registration:**

ClinicalTrials.gov, identifier NCT01051154.

## 1 Introduction

Acute lymphoblastic leukemia (ALL) is the most common pediatric malignancy, accounting for almost one-third of all childhood cancers worldwide, with an incidence of 20-35 per million children under 15 years (1). Mexico City has one of the highest childhood leukemia incidence rates in the world, with 49.5 cases per million children under 15 years of age (2). With the advent of multimodal therapy, the long-term survival rate of children with ALL has improved dramatically and exceeds 90% (3). However, these patients have a higher risk of presenting metabolic alterations during and after treatment, including obesity, dyslipidemia (hypertriglyceridemia, reduced levels of high-density lipoprotein cholesterol (HDL-C), and to a lesser extent, altered levels of total cholesterol and small-dense low-density lipoprotein (LDL-C), hypertension (4), increased adiposity and insulin resistance (5), which suggest an early risk of atherosclerosis and cardiovascular disease (6). These lipemic alterations are a common side effect of treatment with corticosteroids and L-asparaginase in pediatric patients with ALL (7). The side effects of L-asparaginase may be explained by the increase in the endogenous synthesis of very low-density lipoprotein (VLDL-C). In addition, corticosteroid therapy alters lipid and lipoprotein metabolism by increasing hepatic cholesterol synthesis (8). Mexico has the highest prevalence of overweight and obesity in the world (9) and an increased risk of developing metabolic syndrome (MS). Furthermore, several studies have reported that survivors of ALL are at increased risk of MS characterized by obesity (4, 10, 11). Additionally, our group of researchers reported that insulin resistance and body fat were predictive factors of developing MS in survivors of childhood cancer (12). On the other hand, recently, the atherogenic index of plasma (AIP) value has been used not only as an optimal indicator of dyslipidemia but also as a strong novel index for the risk of atherosclerosis and CVD (13).

Different authors have found associations between android fat (abdominal fat located around the trunk of the body) and gynoid fat (gluteal-femoral fat deposited around the hips and thighs) and/or the android/gynoid ratio (A/GR) and different risk factors for cardiovascular disease in children and adolescents (14). Although the etiology of these complications is not completely understood, it has been reported that drug administration, such as doxorubicin, glucocorticoids, and L-asparaginase, is associated with these alterations (5, 6, 15, 16).

Different studies have shown that patients with ALL presents a proinflammatory state at the time of diagnosis and during and after chemotherapy for up to 5 years after the start of treatment, characterized by increased levels of cytokines (IL-1β, IL-6, and TNF-α) (17). In this regard, Sadurska et al. indicated that survivors of childhood ALL are at high risk of developing early atherosclerosis, a multifactorial physiological process that develops over the long term in which dyslipidemia and inflammation are relevant (18).

On the other hand, several studies suggest that dietary supplementation with ω3-LCPUFAs, such as DHA and EPA, can be beneficial for cancer treatment, as it decreases metabolic risk; modulates several aspects of the inflammatory response; decreases inflammatory markers, plasma levels of TGs and LDL-C; and increases HDL-C (19). Nevertheless, studies using ω3-LCPUFAs supplementation in pediatric malignancies are limited (7, 20–22) Some studies have analyzed the effect of fatty acids on weight loss (23) and arm muscle circumference (24) or as adjuvant therapy (22). Previous studies investigating the ω3-LCPUFA effect on hyperlipidemia in children with ALL have reported dramatically reduced TG levels (7, 20, 21)

However, to our knowledge, there are no randomized controlled clinical trials comparing the direct changes in the lipid profile and inflammation markers induced by ω3-LCPUFA supplementation and assessing adherence to treatment by gas chromatography (gold standard) in ALL patients.

Therefore, we present results from a secondary analysis of data obtained in a randomized clinical trial that was previously conducted to evaluate the effect of supplementation with ω3-LCPUFAs on body composition in children with leukemia (under review).

The aim of the present analysis was to examine the effect of supplementation with ω3-LCPUFAs on several cardiometabolic factors in children undergoing treatment for ALL with the hypothesis that ω3-LCPUFAs reduce inflammatory cytokine and TGs levels and increase HDL-C in these patients.

## 2 Materials and methods

### 2.1 Study design

This analysis is part of a randomized clinical trial designed to evaluate the effect of supplementation with ω3-LCPUFAs on body composition in children with leukemia at the end of the remission phase and three months after supplementation, registered in the ClinicalTrials.gov database (clinicaltriasl.gov #: NCT01051154). This study was conducted in accordance with the Declaration of Helsinki, and it was approved by the National Committee of Scientific Research of Instituto Mexicano del Seguro Social (IMSS) and was conducted in the Unit of Research in the Medical Nutrition in a Pediatric Hospital in Mexico City (Approval # 2009-785-107). All parents or legal guardians of the children provided written informed consent prior to study entry.

### 2.2 Patients

We included 72 children with ALL at the Pediatric Hospital of the National Medical Center XXI Century IMSS, National Medical Center, and at the “Gaudencio González Garza” General Hospital, of the “La Raza” National Medical Center. Eligibility criteria were children newly diagnosed with ALL (established according to bone marrow aspirate, immunophenotyping, and immunohistochemistry) at the start of their chemotherapy treatment. Children who had previously been treated with chemotherapy in another institution, those with severe comorbidities, using corticosteroids, taking fish oil supplements during the previous weeks, who were unable to swallow ω3-LCPUFA or placebo capsules, and those who reported allergies to fish intake were excluded. From 72 children with ALL, in this secondary analysis, only 34 children were included (placebo group: 20 patients; ω3-LCPUFA group: 14 patients), due to participants dropping out for different reasons, such as discontinued intervention, death, clinical complications (neutropenic colon), or lack of adherence. We have reported this information in the manuscript of a clinical trial on body composition and omega 3 in children with ALL (currently under review by Clinical Nutrition ID YCLNU-D-23-00112).

### 2.3 Recruitment and allocation

Patients who met all the inclusion criteria and volunteered to participate were randomized in a 1:1 ratio. Selected children were randomly assigned to the placebo group (control) or to the ω3-LCPUFA group (intervention) by a computer-generated list of random numbers using software for parallel groups (Random Allocation Software, http://www.msaghaei.com/Softwares/dnld/RA.zip) (25) The randomization was carried out by balanced blocks of ten children. The unblinded technician supervised the randomization according to the corresponding group. Randomization was blinded for the investigators until the study was concluded.

### 2.4 Intervention

Children received either capsules of ω3-LCPUFAs or placebo capsules. ω3-LCPUFAs were administered in the form of 500 mg soft capsules of natural TGs, made from gelatin, formulated without artificial colors or flavors, molecularly distilled, and of pharmaceutical grade (Nordic Naturals, Inc., Watsonville CA, USA). The omega dose was administered at a rate of 0.100 g/kg of baseline body weight/day. The ω3-LCPUFAs comply with the principles established for fats according to the European Pharmacopoeia Standard (EPS) and according to the Council for Responsible Nutrition (CRN) and the Global Organization (CRNGO). Consequently, ω3-LCPUFAs are a safe product that does not exceed the maximal allowances for contaminants such as peroxides, heavy metals, dioxins, and PCBs. The placebo capsule contained 500 mg of sunflower oil (Progela, S.A. de C.V., México). All capsules contained vitamin E to act as an antioxidant. The odor and appearance of the ω3-LCPUFA capsules and the placebo capsules were comparable, and both were strawberry-flavored to mask their taste. During the study time, all the participants were provided with an oral supplement of the brand Fressenius^®^Kabi, “Frebini Plus” that covered 15% of their total energy expenditure, which was calculated by the Schofield formula. Each 236 ml container of the supplement contained 351 kcal (1491 kJ), 8.9 g of protein, 43.7 g of carbohydrates, and 15.7 g of lipid, and three flavors were employed (chocolate, strawberry, and vanilla). In those patients who presented secondary complications, such as diabetes and pancreatitis, supplementation was contraindicated, and therefore, they were excluded from the study. Children and their parents were instructed to register capsules and oral supplements at the beginning of chemotherapy. During the study period, supplementation was supervised by phone or in the hospital if the patients were staying there by medical personnel.

### 2.5 Compliance

When the patient was discharged, compliance was monitored by the leftover pill count at their next appointment. In addition, the concentration of polyunsaturated fatty acids in erythrocyte membranes was determined before and during intervention with ω3-LCPUFAs. All side effects that the children presented during the intervention with ω3-LCPUFAs, or placebo were documented and registered by one of the researchers.

### 2.6 Procedures

#### 2.6.1 Anthropometry and adiposity

Participants arrived at the medical center between 8:00 and 9:00 am after an overnight fast. Body weight was measured with an electronic scale (TANITA BWB-700, Tanita Corporation, Tokyo, Japan) with the subjects wearing lightweight clothing. Height was measured to the nearest 0.1 cm with a wall-mounted stadiometer (SECA 222, SECA Corp., Oakland Center, Columbia, MD, USA). BMI percentiles for age and sex were calculated according to the Centers for Disease Control (CDC) normative curves using the computer software Epi-info (obesity was defined conventionally as ≥ 95th percentile, overweight as 85^th^ to 94^th^ percentile, and underweight as < 5^th^ percentile) (26). All measurements were made by a nutritionist according to the standard techniques at baseline (both groups, immediately before starting chemotherapy) and upon remission induction. Body fat percentage (BFP) and fat distribution were assessed by dual-energy X-ray absorptiometry (DXA) General Electric Lunar, Prodigy Advance scanner (software version 9.0; GE Medical Systems, Madison, WI, USA) using a low radiation dose (standard 3 µGy). The machine was operated by only one technician. The A/GR was calculated by dividing the fat mass in the android region by the fat mass in the gynoid region.

### 2.7 Analytical methods

Stored serum aliquots were used to determine the lipid profile and cytokines. TGs, total cholesterol, and HDL-C were measured by the enzymatic colorimetric method (SPIN 120 automatic analyzer, Shenzhen, Mindray) with commercially available kits. TGs levels were considered acceptable <75/<90 mg/dL, borderline 75-99/90-129 mg/dL, and high ≥100/≥130 mg/dL for children aged < 10 and ≥ 10 respectively. HDL-C levels were considered low at <40 mg/dL (27); total cholesterol levels were considered elevated at ≥ 200 mg/dL, and the AIP was calculated as the logarithmic transformation of the TGs/HDL-C ratio. AIP values were considered low < 0.1, borderline 0.1-0.24, and high > 0.24 (13). A/GR was calculated by dividing android fat percentage by gynoid fat percentage and values from 85-95^th^ percentile, and >95^th^ percentile have been recently associated with higher metabolic risk in children (28).

Inflammatory markers such as IL-6 and TNF-α were determined in duplicate using high-sensitivity enzyme-linked immunosorbent assay (ELISA) kits, according to the manufacturer’s instructions (R&D Systems, INC., Minneapolis, MN, USA and DSL UK Ltd., Oxon, UK). All assays were carried out in duplicate; coefficients of variation were 8% for ELISAs.

### 2.8 Fatty acid analyses by gas chromatography

Analyses were performed with a 7820A gas chromatograph (Agilent Technologies, Santa Clara, CA, USA) with a flame ionization detector (FID) as described previously (29)

### 2.9 Statistical analysis

The data were analyzed using SPSS 21.0 software for Windows (SPSS, Inc. IBM, NY, USA). Data are presented as the mean ± standard deviation (SD) or as the median (minimal, maximal), according to data (determined by the Shapiro-Wilk test), while categorical variables are presented as frequency (percentages) and were analyzed by Pearson’s chi-square test and Fisher’s exact test as appropriate. The crude significance of within-group and intergroup differences was tested by Student’s t test, paired-samples t test, the Wilcoxon test, or the Mann–Whitney U test, as appropriate. Values of *p* < 0.05 were considered statistically significant.

## 3 Results

**Figure 1:**
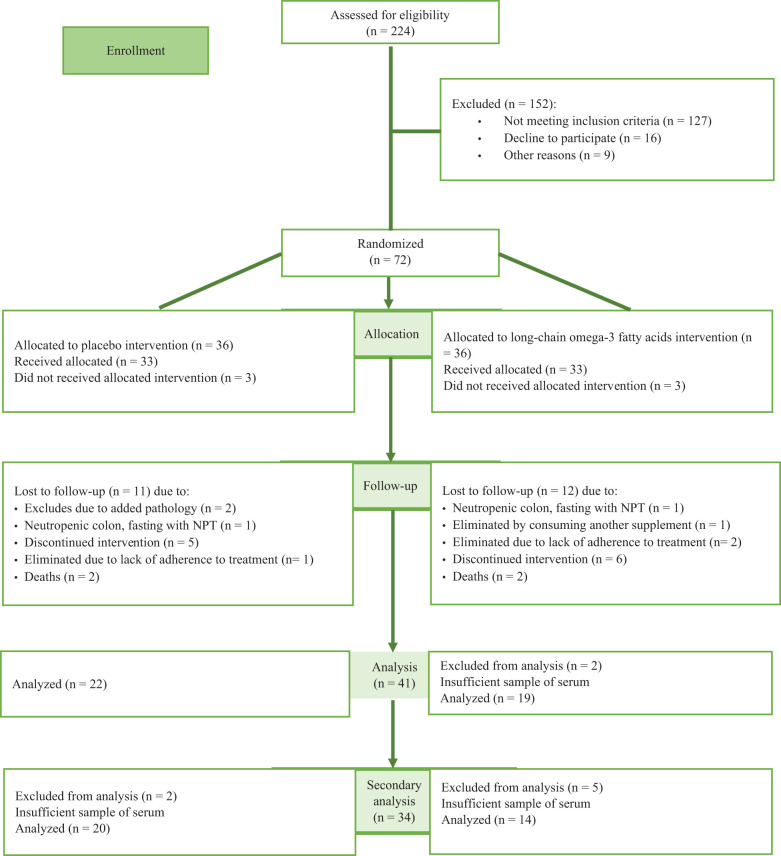
Flow diagram of the progress through the phases of the clinical trial and present analysis.

We summarize the Consolidated Standards of Reporting Trials followed in this study in **Figure 1**. The baseline characteristics of the children who participated in this study were similar across the treatment and control groups, **Table 1**.

**Table 1:** Demographic, clinical, and baseline markers characteristics of children with leukemia.

	Group		*p-value*
	Placebo (n = 20)	ω3-LCPUFAs (n = 14)	
Gender, n (%)			
Male	9 (45)	5 (35.7)	0.728
Female	11 (55)	9 (64.3)
Age, y	7.2 ± 2.8	6.1 ± 2.3	0.271
Body weight, Kg	23.8 (12.5, 57.8)	21.1 (13.5, 41.1)	0.931
Height, cm	122.3 ± 16.6	118.2 ± 16.4	0.479
Clinical parameters, n (%)			
Risk stratification:			
Standard Risk	10 (50)	10 (71.4)	0.296
High Risk	10 (50)	4 (28.6)
Anemia	17 (85)	13 (92.9)	0.627
Thrombocytopenia	17 (85)	13 (92.9)	0.627
Neutropenia	18 (90)	12 (85.7)	1.000
Nutritional status, n (%)			
Eutrophic (BMI pc >5 pc<85)	15 (75)	14(100)	
Overweight ((BMI pc > 85)	2 (10)	0	
Obese (BMI pc > 95)	1 (5)	0	
Undernourished (BMI pc ≤ 5)	2 (10)	0	0.251

Data are presented as mean ± Standard Deviation (SD), at median (minimum, maximum) or as number (percentage). ω3-LCPUFAs, Omega-3 long chain-PUFA; BMI, Body Mass Index; The data were analyzed with an independent-sample t-test or Mann-Whitney U test; Pearson-chi square test or Fisher.

### 3.1 Lipids and inflammatory markers

**Figure 2:**
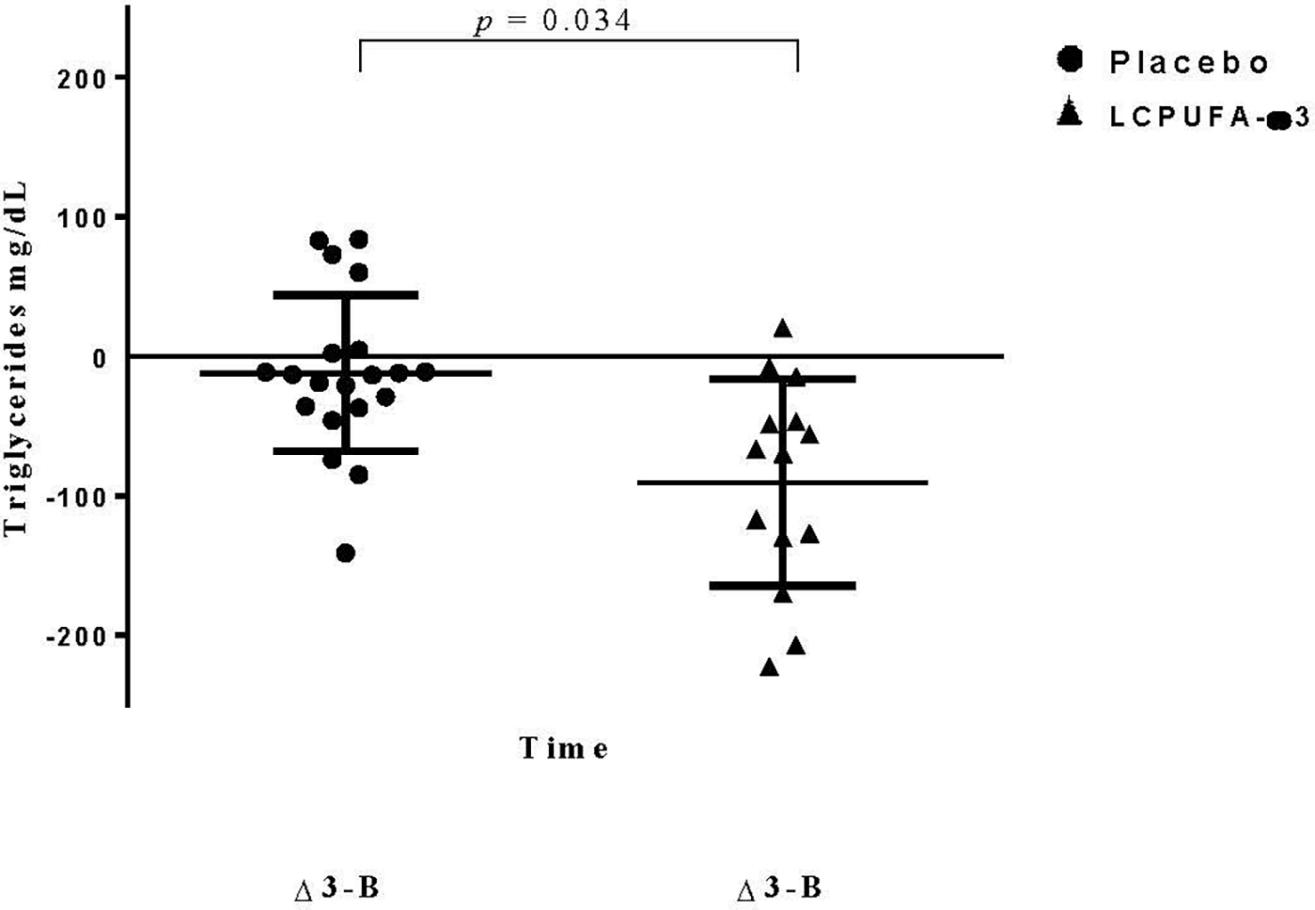
Changes in triglyceride levels. Data are expressed as mean ± standard deviation (SD). Data represent the triglyceride differences during the three months and basal time of treatment. Dependent-sample t-test was performed.

**Figure 3:**
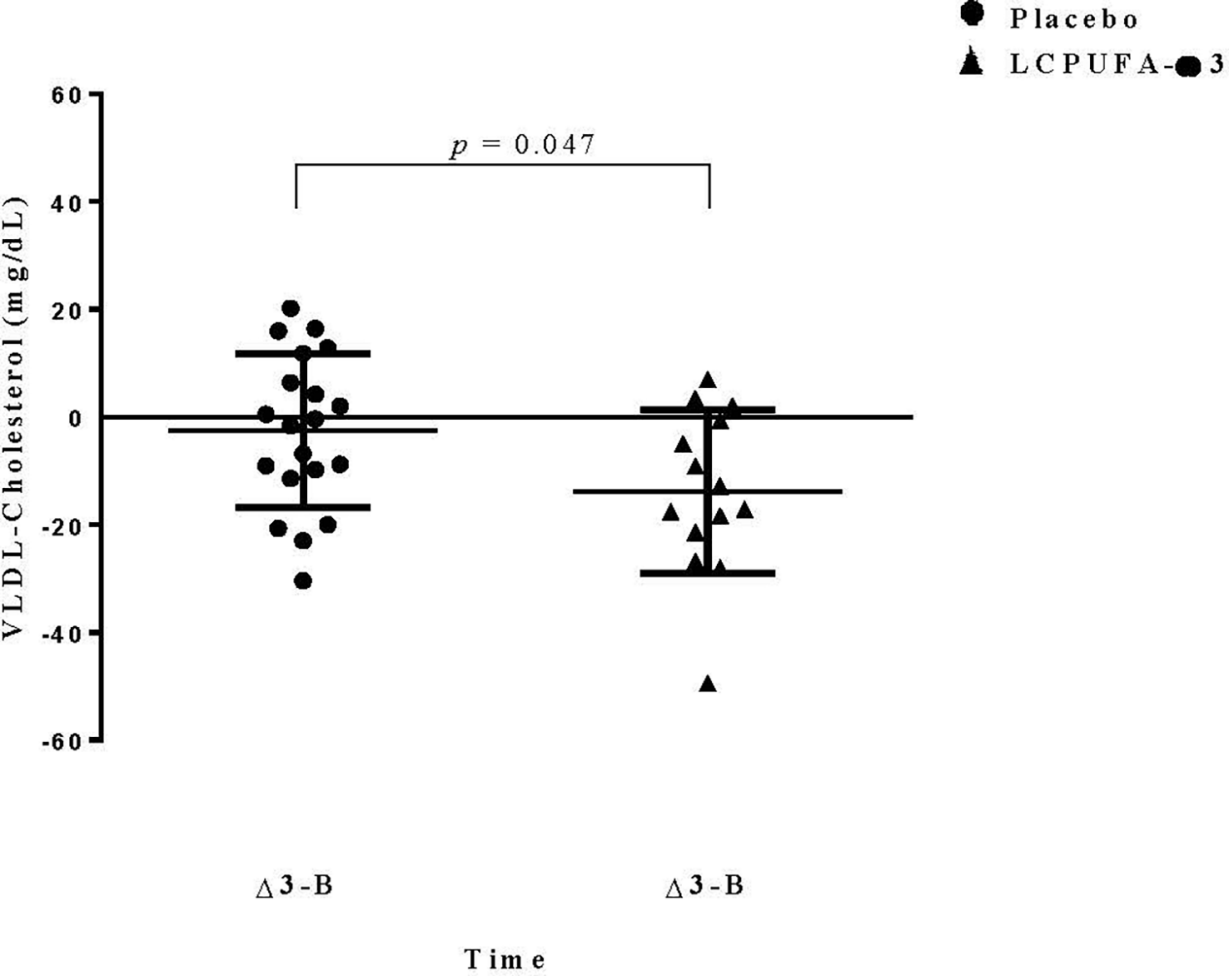
Changes in VLDL-Cholesterol levels. Data are expressed as mean ± Standard Deviation (SD). Data represent the differences in VLDL-C during the three months and basal time of treatment. Dependent-sample t-test was performed.

Blood lipid profiles for each group at baseline and after 3 months of intervention are shown in **Table 2**. At the baseline time point, no significant differences between the groups were observed in total cholesterol, HDL-C, LDL-C, VLDL-C, and TGs. In contrast, we found significantly lower levels of VLDL-C (*p* = 0.039) and TGs (*p* = 0.043) in the ω3-LCPUFA group than in the placebo group at three months. In this sense, after follow-up, we showed that the ω3-LCPUFA group had significantly decreased levels of TGs and VLDL-C between diagnosis and at 3 months of intervention compared with the placebo group (-62.6 ± 20.7 vs. -12.1 ± 12.5; *p* = 0.034; -12.5 ± 4.1 vs. -2.3 ± 2.5 mg/dl, *p* = 0.047, respectively) (**Figures 2**, **3**). In contrast, the total cholesterol concentration was higher at three months (*p* = 0.009) in the ω3-LCPUFA group than in the placebo group. We found that 80% (16/20) and 85% (12/14) of children had hypertriglyceridemia in the placebo group and the ω3-LCPUFA group, respectively, at the time of ALL diagnosis. In addition, when analyzing each group, the VLDL-C concentrations decreased significantly between the time of diagnosis and after 3 months of supplementation with ω3-LCPUFAs (*p* = 0.009), and the number of children with hypertriglyceridemia (*p =* 0.054) tended to be lower. The AIP at 3 months was significantly lower in the ω3-LCPUFA group than in the placebo group (0.49 vs. 0.27; *p* = 0.042); The number of children with an A/GR > 95^th^ percentile was higher in the placebo group than in the ω3-LCPUFA group (47% *p* =0.028).

**Table 2:** Blood Lipid profile at baseline and 3 months of intervention in children with acute lymphoblastic leukemia.

	Placebo (n = 20)	ω3-LCPUFAs (n = 14)		*p-value*	
Total Cholesterol (mg/dL)					
Baseline	120.8 ± 23.6	134.0 ± 23.9		0.118	
Acceptable (<170), n (%)	19 (95)	14 (100)		1.00	
Borderline (170-199), n (%)	1 (5)	0			
High (≥200), n (%)	0	0			
3 Months	129 ± 26.1	155.1 ± 28		0.009	
Acceptable <170)	19 (95)	10 (71.4)		0.135	
Borderline (170-199)	1(5)	4 (28.6)		
High (≥200)	0	0		
*p-value*	0.356	0.017			
HDL-C (mg/dL)					
Baseline	29 (23, 47)	32.5 (22, 48)		0.396	
3 Months	43.5 (24, 94)	50.5 (31, 80)		0.457	
*p-value*	p< 0.0001	0.003			
LDL- C (mg/dL)					
Baseline	84.2 ± 23.4	98.1 ± 33.7		0.160	
3 Months	83.7 ± 26.2	99.7 ± 27.3		0.113	
*p-value*	0.877	0.816			
VLDL- C (mg/dL)					
Baseline	29.9 (17.5, 81)	32.5 (14.5, 71)		0.931	
3 Months	24.8 (11.7, 97)	20.4 (11.6, 40.2)		0.039	
*p-value*	0.167	0.009			
Triglycerides (mg/dL)					
Baseline	149.5 (88, 404)	162.5 (73, 355)		0.931	
(0-9 y)/(10-19y)					
Acceptable (<75)/(<90)	0	1			
Borderline (75-99)/(90-129)	4	1			
High (≥100)/(≥130)	16	12 (85)		0.338	
3 months	124 (59, 487)	102 (58, 201)		0.043	
Acceptable (<75)/(<90)	1 (5)	2 (14)			
Borderline (75-99)/(90-129)	2 (10)	5 (36)			
High ≥100/(≥130) mg/dL	17 (85)	7 (50)		0.075	
p-value	0.156	0.009			
AIP					
Baseline	0.74 (0.41, 1.18)	0.75 (0.31, 1.0)		0.944	
Low < 0.1, n (%)					
Borderline 0.1-0.24, n (%)					
High > 0.24, n (%)	20 (100)	14 (100)			
3 Months	0.49 (0.16, 1.16)	0.27 (0.07, 0.77)		0.042	
Low < 0.1, n (%)	0	2 (14)		0.055	
Borderline 0.1-0.24, n (%)	3 (15)	5 (36)			
High > 0.24, n (%)	17 (85)	7 (50)			
*p-value*	p< 0.0001	0.002		
Total Body Fat (%)					
Baseline	21.6 (9.7, 42.9)	19.9 (11.6, 33)		0.877	
3 Months	27.9 (11.7, 46.5)	22.1 (18.1, 40.3)		0.396	
*p-value*	p< 0.0001	0.001		
(A/GR)					
Baseline	0.54 (0.19, 0.93)	0.54 (0.37, 0.76)		0.610	
<85^th^ Percentile, n (%)	16 (80)	13 (0.93)		
85-95^th^ Percentile, n (%)	4 (20)	1 (0.07)		
>95^th^ Percentile, n (%)	0	0		0.426	
3 Months*	0.88 (0.51, 1.05)	0.77 (0.63, 0.95)		0.189	
<85^th^ Percentile, n (%)	5 (19)	4 (29)		
85 - 95^th^ Percentile, n (%)	5 (19)	9 (64)		
>95^th^ Percentile, n (%)	9 (47)	1 (7)		0.028	
*p-value*	0.001	0.001		

Data are presented as mean ± Standard Deviation (SD), at median (minimum, maximum) or as number (percentage); TC, Total cholesterol; HDL-C, High-density lipoprotein cholesterol; LDL-C, Low-density lipoprotein cholesterol; VLDL-C, Very-low-density lipoprotein cholesterol; AIP, Atherogenic index of plasma; A/GR Android/Gynecoid ratio. *A/GR in the placebo group, one patient was not included due to missing information.

Inflammatory markers in children with ALL from baseline and after three months of supplementation with placebo or ω3-LCPUFA capsules are shown in **Table 3**. Significant differences were observed only in the IL-6 concentration between groups at 3 months (1.4 vs. 0.78 mg/dl, *p* = 0.025).

**Table 3:** Inflammatory markers in children with acute lymphoblastic leukemia from baseline and three months of supplementation with placebo or ω3-LCPUFA capsules.

Inflammatory markers			*p-value*
	Placebo (n=20)	ω3-LCPUFAs (n =14)	
IL 6, mg/dL			
Baseline	2.3 (0.52, 16.5)	1.7 (0.36, 9.6)	0.217
3 Months	1.4 (0.20, 15.6)	0.78 (0.01, 2.31)	0.025
*p-value*	0.037	0.033	
TNF-α, mg/dL			
Baseline	9.1 (2.3, 39.2)	7.6 (1.0, 25.2)	0.478
3 Months	2.3 (0.69, 5.9)	2.0 (0.04, 7.6)	0.959
*p-value*	<0.0001	0.005	

Data are presented as median (minimum, maximum): IL-6, Interleukin 6; TNF-α, Tumor Necrosis Factor.

**Table 4** shows the profile of the polyunsaturated fatty acid composition of erythrocyte membranes in children with ALL at baseline and at three months of treatment. Before supplementation, all fatty acids in the placebo and ω3-LCPUFA groups were similar, and the only significant difference that was observed was for the linoleic acid (LA) concentration (*p* = 0.033), which was consistent at 3 months of supplementation. At three months, the alpha-linolenic acid (ALA) concentration was lower in the ω3-LCPUFA group than in the placebo group (0.48 vs. 0.24 w/w%, *p* = 0.036). After the administration of ω3-LCPUFAs, there were significant and progressive increases in the concentrations of DHA (1.85 vs. 6.0 w/w%, (*p <*0.0001) and EPA (0.28 vs. 1.11 w/w% (*p <*0.0001) between baseline and at 3 months. The concentrations of DHA and EPA in the ω3-LCPUFA group increased by 193% and 289%, respectively.

**Table 4:** Fatty acid composition of erythrocyte membranes in children with acute lymphoblastic leukemia from baseline and three months of treatment with ω3-LCPUFAs.

		Placebo (n = 20)	ω3-LCPUFAs (n = 14)	*p-value*
**LA**	Baseline	12.0 (9.6, 25.2)	10.7 (8.2, 18.8)	0.033
	3 Months	14.5 (9.59, 25.38)	11.7 (9.18, 19.63)	0.030
	*p-value*	0.067	0.064	
**AA**	Baseline	11.4 (0.66, 16.9)	8.7 (2.4, 13.1)	0.259
	3 Months	11.4 (0.80, 15.31)	9.2 (0.87, 11.99)	0.245
	*p-value*	0.232	0.826	
**ALA**	Baseline	0.33 (0.1, 3.3)	0.20 (0.1, 2.9)	0.306
	3 Months	0.48 (0.1, 3.7)	0.24 (0.1, 3.1)	0.036
	*p-value*	0.051	0.41	
**DHA**	Baseline	2.0 (0.08, 3.71)	1.85 (0.39, 2.7)	0.691
	3 Months	1.8 (0.14, 2.71)	6.0 (0.53, 9.14)	< 0.0001
	*p-value*	0.204	0.002	
**EPA**	Baseline	0.32 (0.12, 0.65)	0.28 (0.14, 0.71)	0.545
	3 Months	0.41 (0.08, 0.70)	1.1 (0.30, 2.56)	< 0.0001
	*p-value*	0.619	0.001	

Data are presented as median (minimal, maximal); EPA, Eicosapentaenoic Acid; DHA, Docosahexaenoic acid; ALA, Linolenic acid; LA, Linoleic acid; AA, Arachidonic acid. Significant differences between the two time points were determined by Mann-Whitney U-test and the intragroup by Wilcoxon signed rank test.

## Discussion

In this secondary analysis, we confirmed the effect of ω3-LCPUFA supplementation at a rate of 0.100 g/kg/day on the lipid profile, specifically on the TGs and VLDL-C concentrations during the first 3 months of ALL treatment. We observed a significantly lower concentration of TGs and VLDL-C in the ω3-LCPUFA group than in the placebo group. We found that 82.4% (28/34) of the children had hypertriglyceridemia and low levels of HDL at the time of ALL diagnoses. These results are consistent with previous findings from ALL studies (30–32). In addition, we reported a significantly decreased AIP ratio and IL-6 concentrations between the time of diagnosis and at 3 months in both groups. In contrast, the A/GR increased in both groups over time; the number of children classified in the > 95^th^ percentile was significantly lower in the ω3-LCPUFA group than in the placebo group during the administration of ω3-LCPUFAs. These results confirm that the intervention was effective because of the variation in the rate of change in erythrocyte membranes after supplementation with ω3-LCPUFAs.

There is considerable evidence that EPA and DHA have independent effects on multiple cardiometabolic risk factors, including blood pressure and cardiac function and lipids, as well as anti-platelet, anti-inflammatory, pro-resolving, and antioxidative actions (33). To our knowledge, as we mentioned before, there is a limited amount of information about the effects of ω3-LCPUFAs in a pediatric hematologic population. In addition, neither of these previous studies included the evaluation of the effect of ω3-LCPUFAs on cardiovascular risk factors, such as the VLDL-C levels, AIP, A/GR, and adiposity (body fat percentage), and not just the lipid profile. In this regard, only two studies have evaluated the effect of ω3-LCPUFAs on TGs and total cholesterol in children with ALL (7, 20). Laumann et al., in a pilot study in seven children with ALL (2-10 years), showed beneficial effects on TGs and total cholesterol levels of fish oil capsules containing 2.4 g - 4.8 g of EPA + DHA after the induction phase for six months. The authors reported a decrease in TGs concentrations at days 106 (*p* = 0.025) and 113 (*p* = 0.016) and in the total cholesterol concentration at day 113 (*p* = 0.027) in the intervention group compared to 22 historical controls. Nevertheless, when the authors corrected for one extra PEG-asparaginase dose, the level of significance in both lipid levels was reduced. In this study, the authors did not discuss the bias that the results could present in the intervention group because three patients presented a percentage of compliance of less than 80% (52%, 58%, and 78%). However, they recognized their limited sample size and the disadvantage of using historical controls, and the fact that it was not a randomized parallel-group trial. In our study, both groups were randomized, and the sample size, although small, was higher than that in Lauman’s study.

On the other hand, in a retrospective study, Salvador et al. (7) evaluated the effect of ω3-LCPUFAs (1000-3000 mg/day) in combination with acipimox (250 mg) in 9 patients with ALL with hypercholesterolemia and they observed a decrease in TGs and total cholesterol; however, one patient continued to present hypertriglyceridemia, so Bezalip 200 mg tablets (1 tablet/d) were added to his treatment. Under this therapy, TG levels dropped to the normal range. However, this study had the objective of demonstrating the effectiveness of the combination of omega-3 fatty acids with acipimox as a therapeutic alternative for hypertriglyceridemia and comparing its effectiveness in comparison with the side effects associated with the use of fibrates.

In contrast, we found differences in the total cholesterol between the groups at 3 months of supplementation; however, unexpectedly, the ω3-LCPUFA group presented a higher total cholesterol concentration (*p* = 0.009). These results contrast with those reported by Laumann and Salvador (7, 20). This difference is probably due in part to the maximum doses received of EPA + DHA in Laumann’s and Salvador’s studies (4.8 g and 2.5 g, respectively). Additionally, this result could be partly explained by using “Frebini Plus” milkshakes in both groups, which provided 15.7 g of lipids, and although we did not observe a difference in the groups at the basal time point, we show a tendency for the ω3-LCPUFA group to have a higher level of total cholesterol than that of the placebo group.

On the other hand, we did not find significant differences in HDL-C and LDL-C levels between the groups. In our study, we probably did not find differences since the shake provided in both groups contained 4.3 g of saturated fatty acids which might have influenced the result.

The AIP is a biomarker of atherogenic dyslipidemia that, through non-HDL-C and/or the TG/HDL-C ratio, can predict the risk of a future atherogenic cardiometabolic event from an early age (13, 34–36). Evidence in adults has shown that ω3-LCPUFA administration reduces the AIP. Golzari et al. (37) performed a randomized, double-blind, placebo-controlled clinical trial involving healthy controls and T2DM patients. The AIP was calculated as a proxy measure of atherosclerosis. The baseline AIP value was 0.97 vs. 0.64 after the trial (*p* =.009). In a longitudinal study (38) with 32 overweight and/or obese patients diagnosed with diabetes and hypertriglyceridemia who received fish oil (4.0 g/d) for eight weeks, Souza showed that fish oil supplementation effectively decreased the plasma atherogenic index. In this sense, we found that the omega group had a decrease in the AIP (0.75 vs. 0.27, *p* = 0.002) and in the percentage of patients with a high index (> 0.24) after finishing 3 months of follow-up.

Data on the potential effects of ω3-LCPUFAs on anti-inflammatory actions and cardiometabolic factors in children with ALL are scarce. Nevertheless, there is evidence that ω3-LCPUFAs, reduce inflammatory markers in adult oncology patients (39–41), as explained, n-3 PUFAs reduce promoting cytokines such as IL-1, TNF- α and IL-6 (42). Guo et al. (43), through a meta-analysis on ω3-LCPUFA supplementation and circulating levels of IL-6 and TNF-α in cancer patients, reported that ω3-LCPUFAs can reduce IL-6 and TNF-α levels. We observed significantly lower concentrations of IL-6 in the ω3-LCPUFA group than in the placebo group, additionally a reduction in TNF-α and IL-6 concentrations in both groups between diagnosis and at 3 months.

Based on these results, we should discuss the strengths and limitations of our work. A strength of the study was the use of a prospective cohort, which improved the accuracy of data collection. Another added benefit was the use of controls. In addition, supplementation at a rate of 0.100 g/kg/d (like Bayram et al.) (23) during early phase treatment of ω3-LCPUFAs was effective, and the adherence was measured through the fatty acid profile in erythrocytes (gold standard). On the other hand, body fat mass measurements were obtained using the DXA technique, which is an accurate and reliable measurement in children. A limitation of the study was the small sample size. Although this study has a significantly larger sample than the previous studies by Salvador et al. and Lauman et al. (7, 20),. In addition, in this study, we did not analyze what kinds of food they were eating. An additional limitation was lack of other predictors of atherogenesis such as the measurement of the intimal layer, apo B, etc., as the study was not initially intended for this purpose.

Although ω3-LCPUFAs have a positive effect on cancer patients, in ALL pediatric patients there is no sufficient data for recommendations. As we stated above, available studies from randomized placebo-controlled trials the supplements evaluated have varied in dose, source, time of intervention and kind of supplements used in these of ω3-LCPUFAs (7, 20, 23, 24, 44).

Dyslipidemia and inflammation have some common pathological links, such as obesity and cancer. Recent literature mentions that lipids have a fundamental role in the activation of inflammatory pathways, thus increasing the production of inflammatory cytokines (TNF-α, IL-6 and IL-1), which can promote the interruption of lipid metabolism, especially the reverse transport of cholesterol; this is related to a decrease in HDL-C, which could stimulate compensatory changes, such as the synthesis and accumulation of VLDL-C and hypertriglyceridemia, and ultimately increase cardiometabolic risk (45). On the other hand, the presence of metabolic syndrome in this population increases cardiovascular risk and vascular brain disease as a consequence of premature changes in the arterial wall, including endothelial cell damage (12, 46, 47).

## Conclusion

These findings support the use of omega-3 fatty acids to reduce some adverse cardiometabolic and inflammatory risk factors in children with ALL. Our findings show that an ω3-LCPUFA intervention is feasible, and the results suggest that ω3-LCPUFA supplementation in these patients could help prevent, delay, and/or mitigate the development of dyslipidemia and cardiometabolic conditions that can have a negative impact on them. It is unknown if the effect of omega-3 may last longer than 3 months. We consider that large-scale trials are needed in children with cancer to confirm these results.

## Data availability statement

The raw data supporting the conclusions of this article will be made available by the authors, without undue reservation.

## Ethics statement

This study was approved by the Research and Ethics Committee of the Pediatric Hospital at the Mexican Social Security Institute (2009-785-107). We obtained written informed consent from parents and informed assent from children. Written informed consent to participate in this study was provided by the participants’ legal guardian/next of kin.

## Author contributions

LB-C, designed the study, analyzed data, obtained funding, and prepared the manuscript; SA-M, EJ-A, JM-T and FM-B, contributed to the analysis and interpretation of data and helped prepare the manuscript; ML-A, JM-A performed the analysis and interpretation of data, and helped prepare the manuscript; SD-P, BG, JD-P, AA-B, human resource management, contributed to the analysis and interpretation of data, S-LK, BB-M, AJ-M, ZH-P, EJ-H, LE-H, NN-V, RP-C contributed to the acquisition of data. All authors contributed to the article and approved the submitted version.
